# Environmental effects on constructed wetland microbial diversity and function in the context of wastewater management

**DOI:** 10.1128/spectrum.00229-25

**Published:** 2026-04-21

**Authors:** Sandrine Grandmond-Lemire, Bob Gearheart, Catalina Cuellar-Gempeler

**Affiliations:** 1 Chilkat Indian Village, Haines, Alaska, USA; 2 Cal Poly Humboldt 1285, Arcata, California, USA; 3 Arcata Marsh Research Institute (AMRI), Arcata, California, USA; University of Mississippi, University, Mississippi, USA

**Keywords:** wetland, microbial community, diversity-ecosystem function relationship, water quality, ammonia

## Abstract

Considering temporal and spatial change in biodiversity and ecosystem function (BEF) relationships is critical to predict and manage ecosystem services, especially in human-mediated and impacted ecosystems. We propose that species responses to seasonal change and spatial distributions can act as a laboratory to reveal diversity-function relationships with management implications. This study investigates the relationship between aquatic bacterial diversity and ammonia removal function in a wastewater secondary treatment constructed wetland system. Specifically, we took water samples across a system of six interconnected ponds, from August 2019 to February 2020, at the Arcata Wastewater Treatment Facility (AWTF), in Coastal Humboldt County (California, USA). We used 16S rRNA gene amplicon sequencing to measure bacterial diversity and composition, and an ammonia electrode probe to measure ammonia at the influx and efflux positions of each pond. We found a significant negative relationship between ASV richness and ammonia removal, suggesting that nitrifying and denitrifying bacteria, critical in ammonia removal, are poor competitors, known as a negative selection effect. Based on structural equation modeling, we found that bacterial richness effect on ammonia removal was second to location effects, followed by direct effects of season on richness. We identify taxa associated with function that may influence management strategies, including Planktophila, Legionella, Sulfurimonadaceae, and Sporichtyaceae that thrive in ponds located after chlorination steps. This result enriches the traditional wastewater treatment reactor paradigms to reveal negative BEF relationships that appear within spatial, temporal, and environmental influences. By expanding our views of BEF relationships, we can further unravel how community diversity and composition influence ecosystem processes in natural and humanized systems.

**IMPORTANCE:**

This study sheds new light on how biodiversity impacts ecosystem functions in human-made environments, specifically wastewater treatment systems. By examining bacterial diversity and ammonia removal efficiency across interconnected ponds, we challenge the conventional assumption that more species always lead to better ecosystem performance. The surprising finding that higher bacterial diversity can reduce ammonia removal efficiency (due to competition among key bacteria) offers fresh insights into how microbial communities work. This understanding is critical for improving wastewater treatment and designing systems that maximize efficiency. Moreover, identifying specific bacteria linked to ammonia removal provides practical information for better managing and enhancing treatment processes. By broadening how we think about the relationship between biodiversity and ecosystem function, this study offers valuable tools for both scientists and environmental managers working to balance human impact with ecosystem health.

## INTRODUCTION

Biodiversity and ecosystem functioning (BEF) relationships have been an area of inquiry for community and ecosystem ecologists for the past three decades (1–3). The literature generally supports positive BEF relationships, with abundant evidence focusing on primary productivity or biomass accumulation as the central function (3–5). The BEF field has now been extended to understanding the impact of climate change and human activities on biodiversity for terrestrial, marine, and aquatic habitats, as well as serving as an urgent call for conservation to maintain ecosystem functions (1, 6, 7). However, this positive BEF trend is not unique, with field studies demonstrating diverse trends in biodiversity and function relationships (8–10). Importantly, we have a poor understanding of how non-positive BEF relationships respond to climate and human-induced change, especially for ecosystem functions like degradation (due to negative selection effects [11–13]), pathogen defense (due to dilution effects [14, 15), and grassland productivity (due to priority effects [16]). Instead of searching solely for a generalized BEF relationship, we should unveil how different BEF relationships respond to environmental change and move toward a more dynamic view of biodiversity-function relationships (17, 18).

A dynamic view of diversity is well supported by our understanding of assembly processes, yet the functional outcomes remain less clear (19–21). Processes that may influence diversity and function include abiotic filtering, species interactions, and spatial arrangement of habitat patches (17, 19). Abiotic factors along a gradient of increasing stress can reduce diversity through habitat filtering, favoring species with tolerance traits that enable them to maintain viable populations (22) while also selecting the functional traits of the community (23, 24). In turn, species interactions can exclude species and their function through mechanisms like competitive exclusion but can also modify the functional output of coexisting species through co-regulation (25, 26). When considering communities interconnected in space (metacommunities), it is key to consider how space can influence diversity via dispersal limitation and heterogeneity (27–29). Overall, predicting the combined effects of these mechanisms is challenging, as both diversity and function can respond to the same underlying variables (17). We can address this challenge by leveraging BEF relationships along environmental gradients to make quantitative predictions (7, 30–32). Such an approach would allow us to address a key remaining question: whether environmental conditions and assembly processes exert a stronger influence on function than diversity itself. This suggests that the environment has non-random effects on species contributions to function (33, 34), not through shifts in population density but via per capita functioning (32).

This study focuses on the aquatic bacterial communities within the Arcata Wastewater Treatment Facility (AWTF), which is composed of oxidation ponds and constructed treatment wetlands used to remove waste and toxins from wastewater before releasing it into the Humboldt Bay. The AWTF is composed of three phases of constructed ponds categorized as a free water hydraulic design (Supplemental methods). The ponds are connected in series to facilitate wastewater processing, with particular focus on ammonia removal. Ammonia is one of the principal pollutants found in wastewater, originating from industrial and house-cleaning chemicals, amino acid products, and urine in sewage (35) with consequences for algal blooms, effluent quality, and public health (36, 37). While traditional wastewater systems rely on costly energy inputs to function (38, 39), constructed wetlands provide a series of habitats where macrophyte and microbial communities perform metabolic processes that remove ammonia from the untreated or previously treated wastewater (40–43). In oxidation ponds, for example, algae and macrophytes can uptake ammonia directly, while providing physical substrate, carbon sources, and oxygen to support bacterial nitrification (44, 44–47). Bacteria have emerged as a key driver of nitrogen removal, particularly in free open surface water designs like AWTF due to their metabolic diversity (48).

The metabolic pathways central to ammonia removal from wastewater are nitrification, denitrification, and direct uptake by algae, aquatic macrophytes, and bacteria. Nitrifying bacteria, like *Nitrospira* and *Nitrosomonas* (49), metabolize the ammonia into bioavailable nitrates, which photosynthetic organisms require for growth, including primary producers like plants and phytoplankton (50). While primary producers can utilize ammonia as a nutrient source, wastewater contains it in excess, and plants or phytoplankton instead require a balanced nitrate-to-ammonia ratio to optimize ammonia uptake efficiency (51). Following nitrification, a large portion of the nitrate is reduced to molecular nitrogen and released into the atmosphere by denitrifying bacteria, like *Paracoccus denitrificans* (37), thus completing ammonia removal from the system. Even though portions of these processes occur in sediments and the sediment-water interface (52, 53), they are routinely studied in bacterial communities found in the water column, which, due to its accessibility, can be considered an indicator of system performance (54, 55). Importantly, bacteria responsible for ammonia removal require large and complex enzymes, tend to grow slowly, and are poor competitors, raising questions of their persistence in the system and maintenance of their critical ecosystem function in wastewater-impacted habitats (56–58).

Bacteria-driven ammonia removal may be influenced by seasonal environmental change and the spatial distribution of the wastewater treatment wetlands. These are key distinctions between traditional wastewater treatment systems and constructed marshes. Traditional wastewater systems consist of a fixed number of reactors, largely isolated from external environment conditions, while constructed wetlands feature spatially explicit interconnected ponds that facilitate microbial community mixing via water flow and are directly influenced by surrounding environmental conditions. This makes constructed wetlands an intriguing system to evaluate the effect of location and season on bacterial diversity and ammonia removal.

We consider four hypotheses that can explain the emergence of negative (Hypotheses 1 and 2), positive (Hypothesis 3), and neutral (Hypothesis 4) BEF relationships. First, different habitat conditions across the constructed ponds can generate heterogeneity and dispersal limitation that may support ammonia removal via regional coexistence of otherwise poor competitors (Hypothesis 1). Second, we hypothesize that season could result in habitat filtering due to low temperatures in winter, benefiting cold-tolerant taxa that may or may not be contributors to ammonia removal (Hypothesis 2). A third alternative would result in a positive BEF relationship, where bacteria contributing to ammonia removal benefit from increased diversity that supports facilitative or mutualistic relationships between bacterial taxa, via cross-feeding (59, 60) or complementarity (6, 7, 61, Hypothesis 3). Lastly, taxa could be functionally redundant and achieve ammonia removal efficiently, regardless of their diversity or composition (known as functional redundancy [62–64], Hypothesis 4). Understanding the mechanisms that sustain taxa capable of ammonia removal will enable us to more accurately predict changes in diversity and function across environmental and biological gradients.

The central goal of this study is to investigate whether species responses to seasonal environmental change and habitat spatial distributions reveal diversity-function relationships with management implications. Specifically, we ask how location and season influence the relationship between bacterial communities and their function in the context of ammonia removal within the AWTF. Based on the specific enzymatic investment requirements of nitrification and denitrification processes, we expect to find a negative relationship between bacterial diversity and ammonia removal. We also hypothesize that bacterial diversity will have a stronger effect on function than environmental conditions and spatial relationships because of the strong metabolic component of this process. To understand the underlying processes, we identified bacterial taxa associated with ammonia removal and explored their potential functional and coexistence traits. We assess community-level responses to season and location in an effort to propose strategies that maximize ammonia removal from wastewater before the bay discharge point. By expanding our understanding of microbial BEF relationships in these wastewater treatment wetlands, we can further unravel dynamics in natural and humanized systems with the goal of predicting, managing, and sustaining adequate ecosystem functions.

## MATERIALS AND METHODS

**Fig 1:**
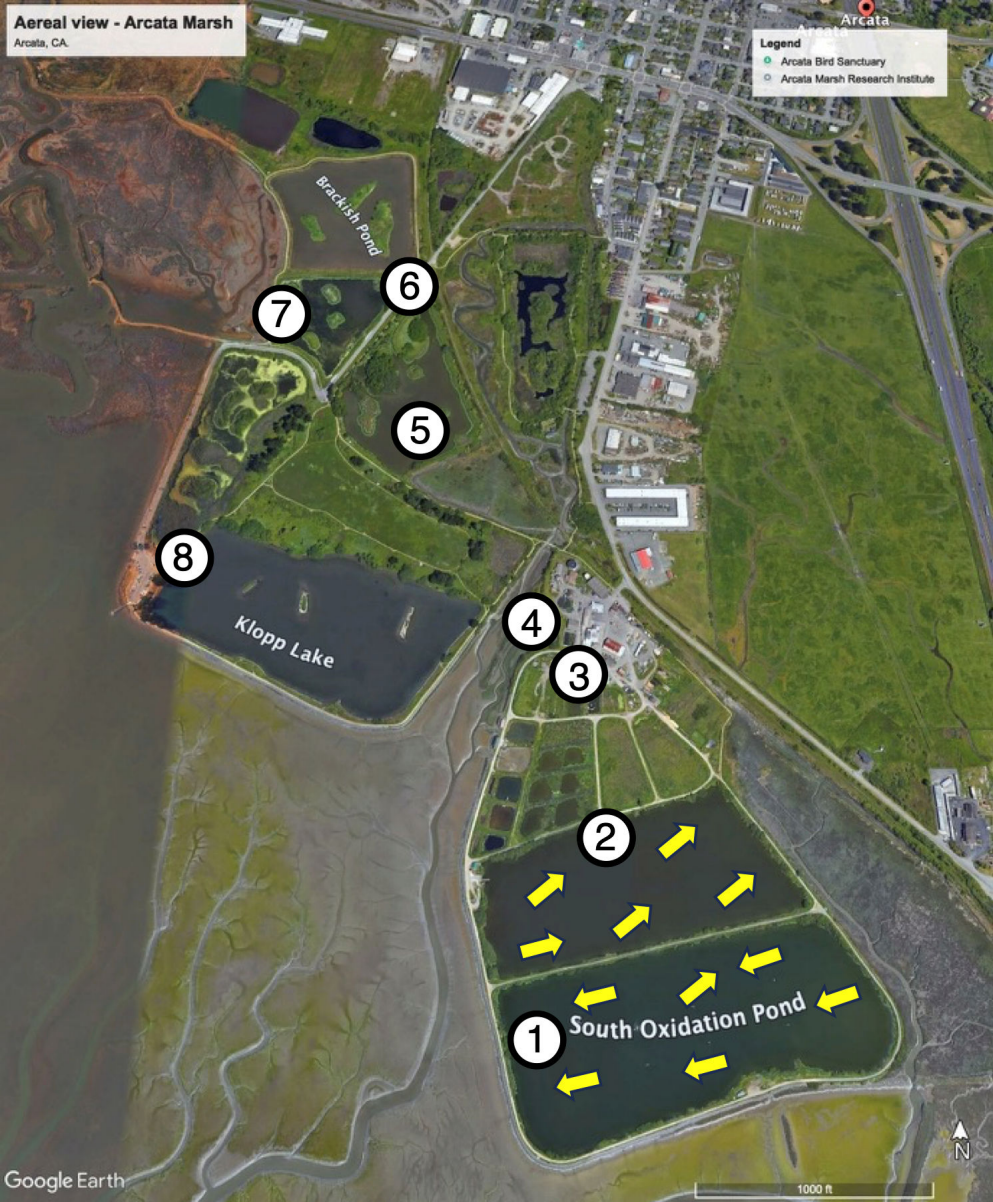
Map of the study site and sample locations. Numbers indicate sample locations at weirs that access the influent and effluent for each pond. For oxidation ponds (1, 2), yellow arrows represent sampling locations as distributed along the water flow from influent to effluent. The samples from the treatment marshes (3), EW influent (4), Allen pond (5), Gearheart pond (6), Hauser pond (7), and Bay discharge (8) were taken at weirs marked by the numbers. Map obtained from Google Earth (August 2020, version 9.0). See Supplemental methods for additional descriptions of the ponds and the treatment train. Imagery Google Earth (2026).

This study was conducted from October 2019 to February 2020 at the Arcata Marsh located in Coastal Humboldt County (California USA). These constructed wetlands are composed of ponds connected in series: oxidation ponds 1 and 2, parallel treatment wetlands, Allen Pond, Gearheart Pond, and Hauser Pond (Fig. 1; Supplemental material). There is a chlorination step before oxidation pond 1, one after the parallel treatment wetlands, and a last one before the Bay discharge point. Ponds vary from open water (oxidation ponds) to semi-vegetated (Allen, Gearheart, Hauser ponds, Supplemental material).

This region experiences mild seasonal changes throughout the year, which are categorized as a wet and dry season. The rainy season averages 100 cm in winter (December to February), while the dry season (May to September) experiences as low as 12 cm of rain (Table S1, National Weather Service). However, the winter season was unusually dry in 2019, compressing the seasonal periods (Table S1). Based on these parameters, we consider our study to encompass three seasons: autumn (October 2019), winter (November 2019–early January 2020), and spring (late January and February 2020). We sampled the water column every 2 weeks to account for these seasonal periods for a total of eight sampling dates. Other abiotic parameters are relatively consistent over time. For example, pH, which is consistently neutral to slightly acidic according to over 20 years of AMRI records (average 7.2, range 6.5–8.2 [65, 66]).

Sampling points were chosen to profile how the microbial community and water quality changed as water entered and exited each pond and included all seven wetland sites in the system (Fig. 1). At each sampling date, we took a total of 17 samples that included five samples on each of the two oxidation ponds, two samples from the treatment marshes, two samples from each of the three enhancement marshes, and one bay discharge sample. Samples from the two large oxidation ponds were obtained by boat and encompassed more samples to obtain a reliable profile of their bacterial community and water quality. For the treatment marsh samples, the effluent weir from oxidation pond 2 was selected as a representative treatment marsh influent sample, and the effluent was collected as a composite sample at a pre-existing pipe that served as a common effluent for the treatment marshes. The following three enhancement wetlands were sampled via weirs and pump stations chosen to match sampling points used regularly for monitoring conducted by the Arcata Marsh Research Institute (AMRI). For each sampling site, we recorded the water temperature and dissolved oxygen (DO) using a Hannah multiparameter probe at three depths (surface, 30 cm, and 90 cm deep) for each location (65).

Our study focuses on water column bacteria as key representatives for ammonia removal, following the approach used in numerous previous studies (40, 67–69). As we mentioned earlier, important portions of the nitrogen cycle occur in the sediment and water-sediment interface (53, 70), yet the water column can be a dynamic (71), and environmentally sensitive (72) indicator with a diverse heterotroph community in a relatively homogeneous habitat (73). In this system, the aquatic habitat offers a much larger volume for nitrogen transformation than biofilms and the best point of comparison across heterogeneous ponds.

### Collection and filtration

Water samples were collected with autoclaved sterile Nalgene 1L bottles, stored in ice, and transported to the field laboratory for further processing. For each water sample, 400 mL was filtered using sterile analytical filter funnels with a pore size of 0.45 µm to minimize clogging from the high algae concentration. Then, the flow-through was collected in a sterile filter flask and filtered a second time through using a pore size of 0.22 µm to capture smaller-sized bacteria. All processed filters were preserved in 1.5 mL microcentrifuge tubes with 1 mL of DNase/RNase buffer Zymo Shield (Zymo, California) and stored at −80° C until ready for extraction.

The remaining sample was used to measure ammonia and nitrate concentration using an Orion meter and the ISE high-performance ammonia electrode with substrate-specific buffer and 1,000 ppm standard solutions (USAbluebook, Illinois). The ammonia test was done with an ammonia probe and calibrated with 100 ppm, 10 ppm, 1 ppm, and 0.1 ppm standards.

### DNA extraction, sequencing, and bioinformatics

We used ZymoBIOMICS DNA/RNA extraction kits (Zymo, California) designed for mixed microbial community samples as described by the manufacturer. The resulting DNA was sequenced using the Illumina MiSeq platform at Argonne National Laboratories. Briefly, the 16S rRNA genes were targeted using archaeal and bacterial primers 515F and 806R, aiming for the V4 region of *Escherichia coli* in accordance with the protocol described in similar previous work (74, 75) and applicable by the Earth Microbiome Project (https://earthmicrobiome.org/protocols-and-standards/16s/). The raw DNA sequences have been submitted to the NCBI Sequence Read Archive (SRA) and are accessible under accession number PRJNA1426766.

Raw sequences were demultiplexed using idemp and then quality-filtered and clustered using the DADA2 R package (version 1.26, [76]). Demultiplexed data matching Phi-X reads were removed using the SMALT 0.7.6 akutils phix_filtering command. Chimeras were removed using VSEARCH 1.1.1 (77). Sequences were clustered into amplicon sequence variants (ASVs) using Greengenes version 13.5 (78) to determine taxonomy. To assign taxa to the ASV table, we used the function dada() at the 97% similarity. Using the Phyloseq package (version 3.9, [79]), we removed ASVs assigned to Archaea, Mitochondria, unassigned taxa, and those shorter than 1,800 number of reads. We used Cumulative Sum Scaling to normalize the resulting reads using the cumNorm() function from the Metagenomeseq package (version 3.19, [80]).

### Data and statistical analysis

All analyses and calculations were completed in R (version 1.4.1564, [81]), and all graphs and plots were constructed with the package ggplot2 (version 2.5.2, [82]) using the viridis package for colorblind-friendly palettes (83). Custom scripts are available in the CGLab GitHub page (https://github.com/catalicu/env_marsh_bef).

We calculated diversity metrics from the processed ASV table to obtain richness and Pielou’s evenness (84) using functions specnumber() and diversity() in the vegan package (version 2.6-8, [85]). We averaged the replicate samplings conducted in oxidation ponds 1 and 2 per date, to account for their large size and maintain equal sampling across ponds. To establish a metric of microbial function, we calculated the delta ammonia concentration of ponds for each sample date by subtracting the effluent ammonia concentration of each location from its influent concentration (equation 1).


(1) $$\Delta {NH}_{3}=[{NH}_{3}{]}_{in}-[{NH}_{3}{]}_{out}$$

We used a model selection approach to determine the role of richness, season, and location in driving ammonia removal (delta ammonia) as calculated using equation 1. We included a linear model with richness as a fixed factor and sequentially added season and location as additional fixed factors. We included interactions between season and richness, and season and location, but only included the additive effect of each model instead of full interactions (richness*season*location) to avoid collinearity effects. We compared linear models using the Akaike Information Criterion (AIC) and Bayesian Information Criterion (BIC). Because both criteria indicated the same patterns, we only used AIC in subsequent analyses. We evaluated the best-performing model using a Wald test with the function Anova() from the car package in R (86) and by comparing the best-performing model to the null model using the anova() function from base R.

To reveal diversity and function-independent responses to location and season, we used a similar model selection approach as described above, using the AIC as selecting parameter. We included linear models with season as an explanatory factor and GLMM with season as a fixed factor and location as a random factor. We conducted a Wald test on the best-performing model and confirmed our findings by comparing models as described above.

We used structural equation modeling (SEM) to evaluate the simultaneous influences of location and season on richness and delta ammonia while accounting for BEF relationships (87). SEM can be used in ecology to estimate the relationships between multiple dependent and independent variables and better model predictor variables (88, 89). Our *a priori* model (sensu [90]) is based on the above GLM and ANOVA approaches and includes location and season as exogenous variables, while richness and delta ammonia are endogenous variables. We used the function sem() from the package lavaan in R (version 06-17, [91]) to build the models. Categorical parameters (location, season) were encoded as nominal ordered values to preserve sequence, and numerical parameters (richness, delta ammonia) were scaled and centered at zero. For this analysis, only sites that allowed us to match diversity and function were included. Because of this small sample size (*n* = 33), several of the more complex models were just-identified (had zero degrees of freedom), resulting in saturated solutions that could not be evaluated with global model fit indices. To address this limitation, we adopted a progressive model simplification approach (92–96), sequentially removing paths and predictors while retaining biologically meaningful structure, with the goal of obtaining models with positive degrees of freedom.

We used a model selection approach to identify the best fit (97, 98) using AIC and BIC to compare across unsaturated models. To compare the covariance structure in the model with the covariance matrix of the data, we used five goodness-of-fit methods: χ^2^, root-mean-square error of approximation (RMSEA), standardized root mean squared residual (SRMR), comparative fit index (CTI), and Tucker-Lewis Index (TLI) (99, 100). We used the psem function from the piecewiseSEM package in R to estimate the significance of individual factors within our models from independent regression estimations (101). We reproduced this analysis by including precipitation and temperature with or without location to evaluate whether the seasonal effect was driven by one of these parameters independently, yet these models were also just-identified and were therefore excluded from further analysis. The magnitude and direction of coefficients were assessed with standardized path coefficients evaluated with Wald z-tests (102).

We assessed the variation of environmental parameters during our study and across the AWTF. The variation of temperature and precipitation is evaluated with linear models since the connectivity between ponds is less significant for these parameters. Season and location were included as factors. Temperature and precipitation were regressed against one another to assess co-variation and, since we found correlations, only the direct effect of temperature was assessed on diversity metrics using linear models. To determine whether the total concentration of ammonia changed with season and location, we used a GLMM with a Gaussian distribution that included season as a fixed factor, and location as a random factor. We evaluated the significance as indicated above for other GLMMs in this study.

To consider the influence of community composition on BEF relationships, we used a perMANOVA to establish the effects of season and location on microbial composition with the function adonis2() from the package vegan. We further established the independent contribution of season and location using the pairwise.adonis() function (103). For this analysis, we used taxa with 100 or more reads to focus on ASVs that were consistently represented in our data set. We then assessed whether these patterns were driven by differences in community similarity by calculating the Bray-Curtis dissimilarity index and running a multivariate analog to Levine’s homogeneity of variances, using the function betadisper() from the package vegan. We ran a non-metric multidimensional scaling (NMDS) with three dimensions to illustrate these patterns.

We established the taxonomic groups underlying community and functional patterns using a combination of statistical modeling and heatmap imaging. We used a linear model with delta ammonia as the dependent variable and ASV relative abundance as the explanatory variable to identify potential taxa contributing to function. We focused on the 350 most abundant ASVs, accounting for 75% of the abundance in our data set and with more than 1,600 reads in total. We adjusted *P* values using the Benjamini and Hochberg (1995) method to account for multiple testing (104). We selected taxa with significant associations with ammonia removal and performed a GLMM with Gaussian distribution, season as a fixed factor and location as a random factor, to establish how these taxa abundances vary across our data set. We also conducted this modeling at the taxonomic level of family. Significance and corrections were performed as above. To illustrate broader patterns in community composition shifts across location and season, we used the function geom_tile() in the ggplot2 package.

## RESULTS

After quality filtering, we obtained 3,945,058 reads distributed across 137 samples with an average of 29,007 reads per sample (±6,864), and clustered in 8,702 ASVs from all wastewater samples combined throughout the treatment plant for the duration of the study. The taxa belonged to 47 Phyla, 438 Families, and 753 Genera from the Kingdom Bacteria, with only 9 attributed to Archaea. In the following paragraphs, we will show the broader BEF relationship, the effect of location and season on ASV diversity and ammonia removal function, the environmental conditions potentially underlying the effect of location and season, and the effect of community composition, while identifying taxa associated with ammonia removal.

### Overall BEF relationship

**Fig 2:**
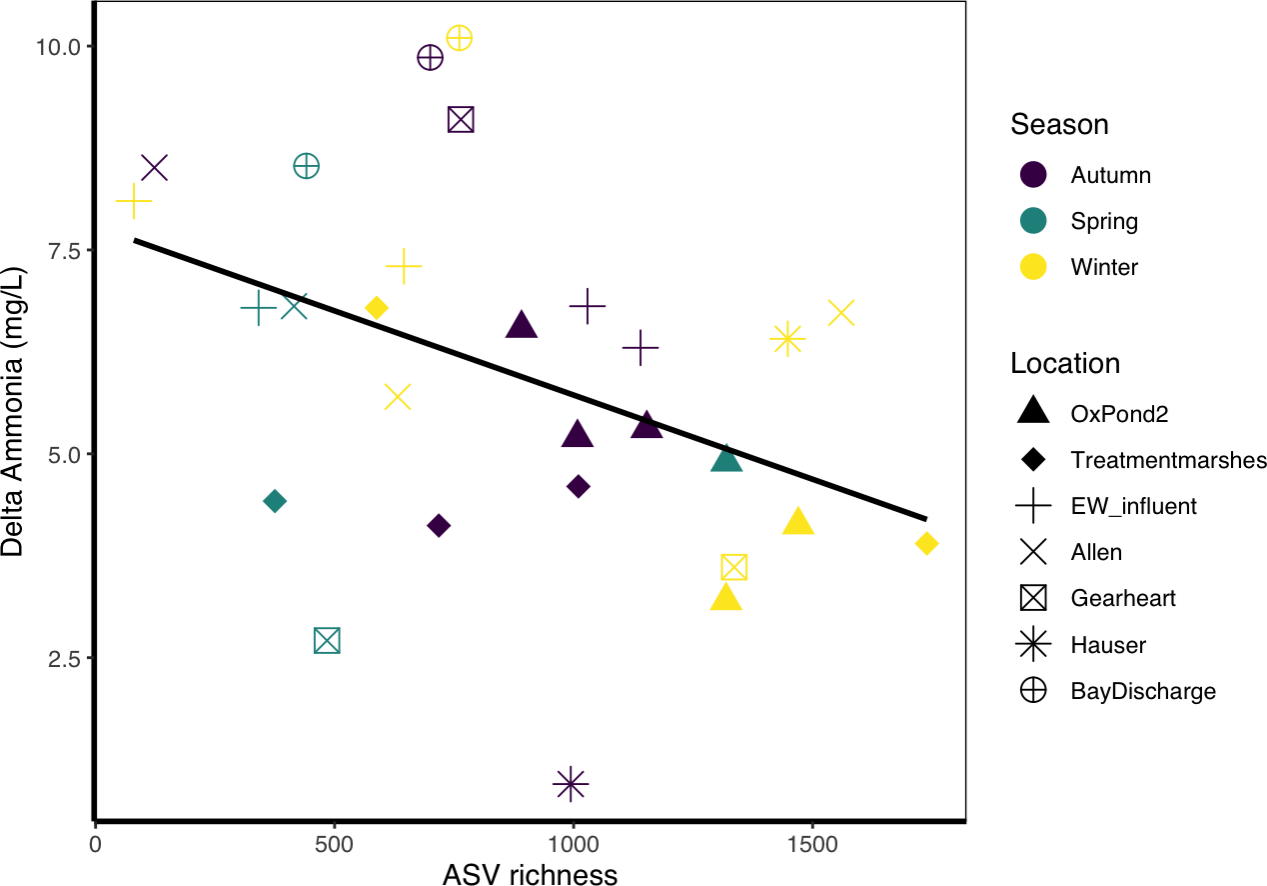
Negative BEF relationship between diversity and the average change in ammonia for each pond at each date. The line represents the linear relationship between richness and delta ammonia.

We found a significant negative BEF relationship between richness and function when accounting for all samples across the wastewater treatment plan for the duration of the study (Fig. 2). The best-performing model included richness, season, and location as explanatory factors, as well as the interaction between richness and location (Fig. 2; Table S2a). Location was the only significant factor after the Wald test (F_6_ = 3.800, *P* = 0.023). Despite richness not reaching statistical significance after the Wald test (F_1_ = 1.953, *P* = 0.187), its inclusion markedly improved model performance relative to the null model (F_15,12_ = 5.359, *P* = 0.028). Samples at the lowest range of the richness gradient had, on average, 101.5 ASVs and corresponded to the removal of 8.305 mg/L ammonia. In contrast, samples at the highest range of the richness gradient had, on average, 940.68 ASVs, but only accounted for 5.315 mg/L ammonia removal. There was also a significant relationship between delta ammonia and species evenness (F_13,14_ = 8.714, *P*-value <0.001, adjusted *R*^2^ = 0.978, Fig. S1a). The best-performing model included evenness and location as fixed factors (Table S2b).

### Effects of location and season

**Fig 3:**
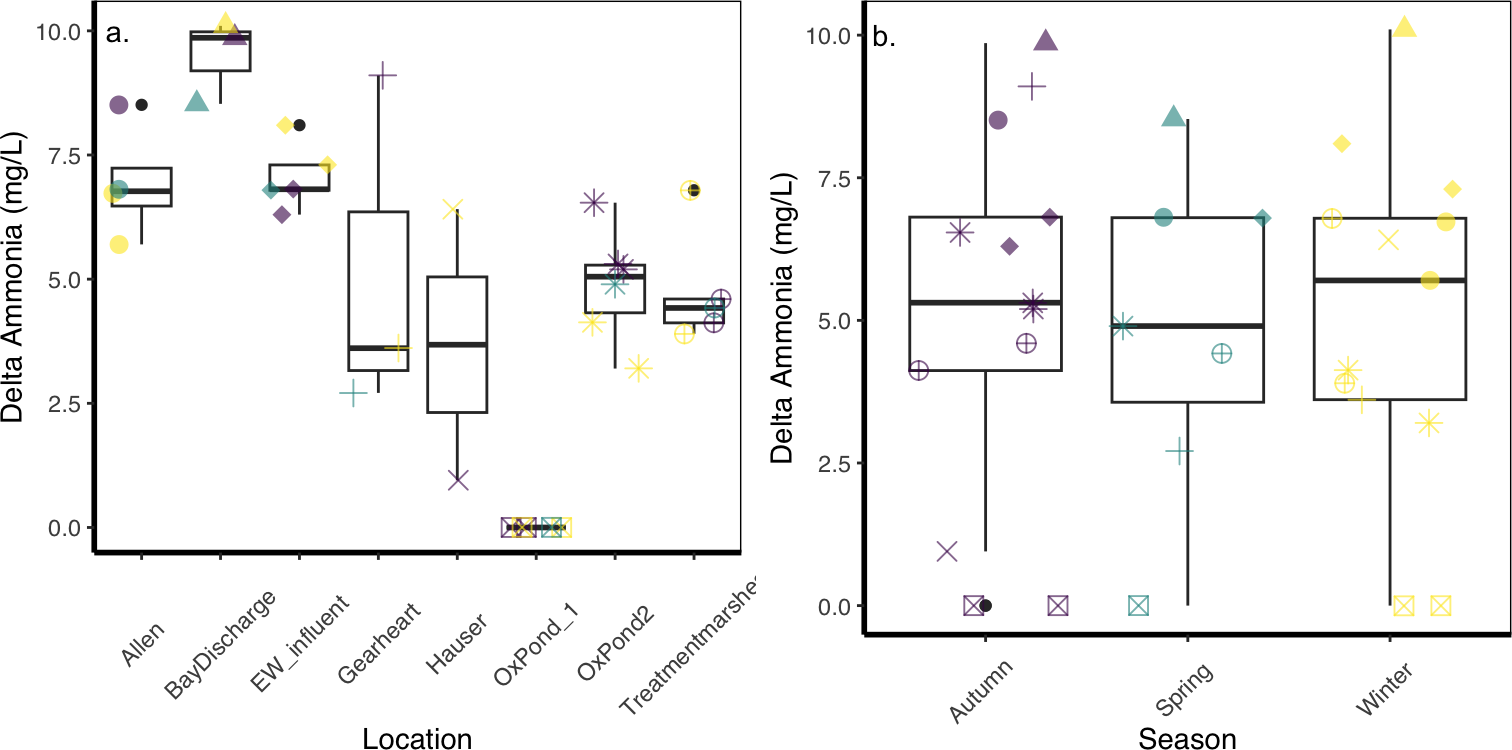
Effects of location (**a**) and season (**b**) on the change in ammonia concentration. In (**b**), locations are ordered from the input to the bay discharge point. See Fig. 2 for legend details.

The function of ammonia removal (delta ammonia) responded to location but not to seasonal change (Fig. 3). The best-performing model included location and season to explain delta ammonia data (Table S3). The Wald test revealed a significant effect of location (F_7_ = 48.005, *P* < 0.001) and an interaction between location and season (F_13_ = 5.370, *P* = 0.005), but no effect of season (F_2_ = 2.685, *P* = 0.116). The model performed better than the null model (F_10,32_ = 18.474, df = 1, *P* < 0.001). Ammonia increased and then decreased along the series of treatment marshes, with higher values in Allen Pond and at the bay discharge point (Fig. 3a). Although season was included in the best-performing model, its overall effect may be explained by its interaction with location, instead of its direct effects on delta ammonia (Fig. 3; Table S3).

**Fig 4:**
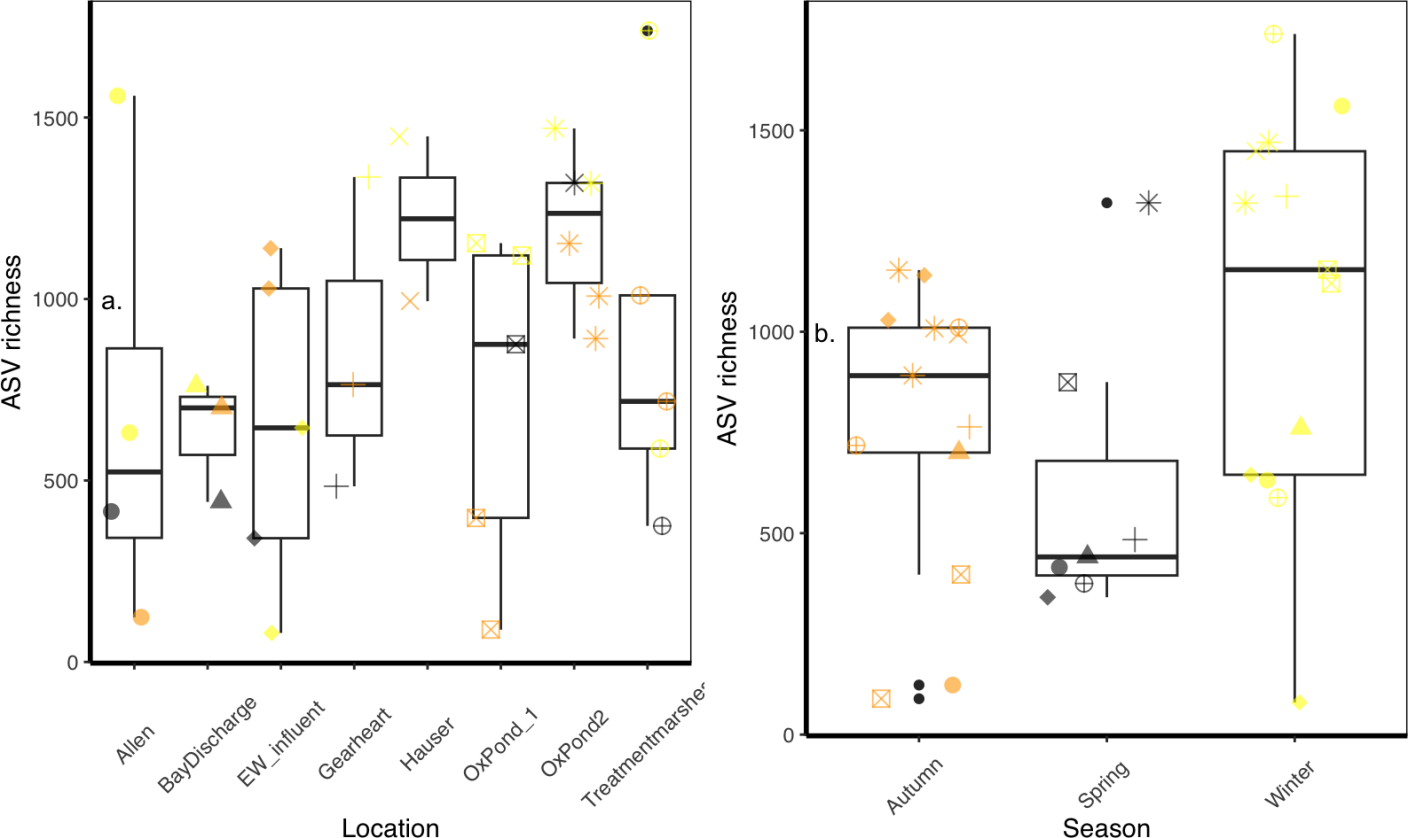
ASV richness responses to (**a**) location and (**b**) season. Datapoints represent individual samples at each sampling event. Colors represent seasons in (**a**) while shapes represent locations in (b). Oxidation pond data were averaged to account for their large sizes and multiple sampling.

ASV richness responded to season and, only weakly, to location. Season had a significant effect on richness (χ^2^ = 8.1269, df = 2, *P* = 0.0172). Winter had the highest ASV numbers (1,256 taxa), declining toward spring (603 taxa, Fig. 4). The contribution of location was not significant, even though the best-performing model included the random variable (Table S4a). Notably, oxidation pond 2 and Hauser had the highest mean richness, while the lowest averages were found in Gearheart and Bay discharge points. These low points corresponded with the chlorination steps in the treatment train. Allen marsh had the highest variability in richness but was not significantly different from the other locations (Fig. 4). Evenness showed a similar pattern with Season as the main contributing factor, but the best-performing model was a linear model with only Season as a fixed factor (Fig. S1; Table S4b, Wald test: F_2_ = 6.1714, *P* = 0.004).

**Fig 5:**
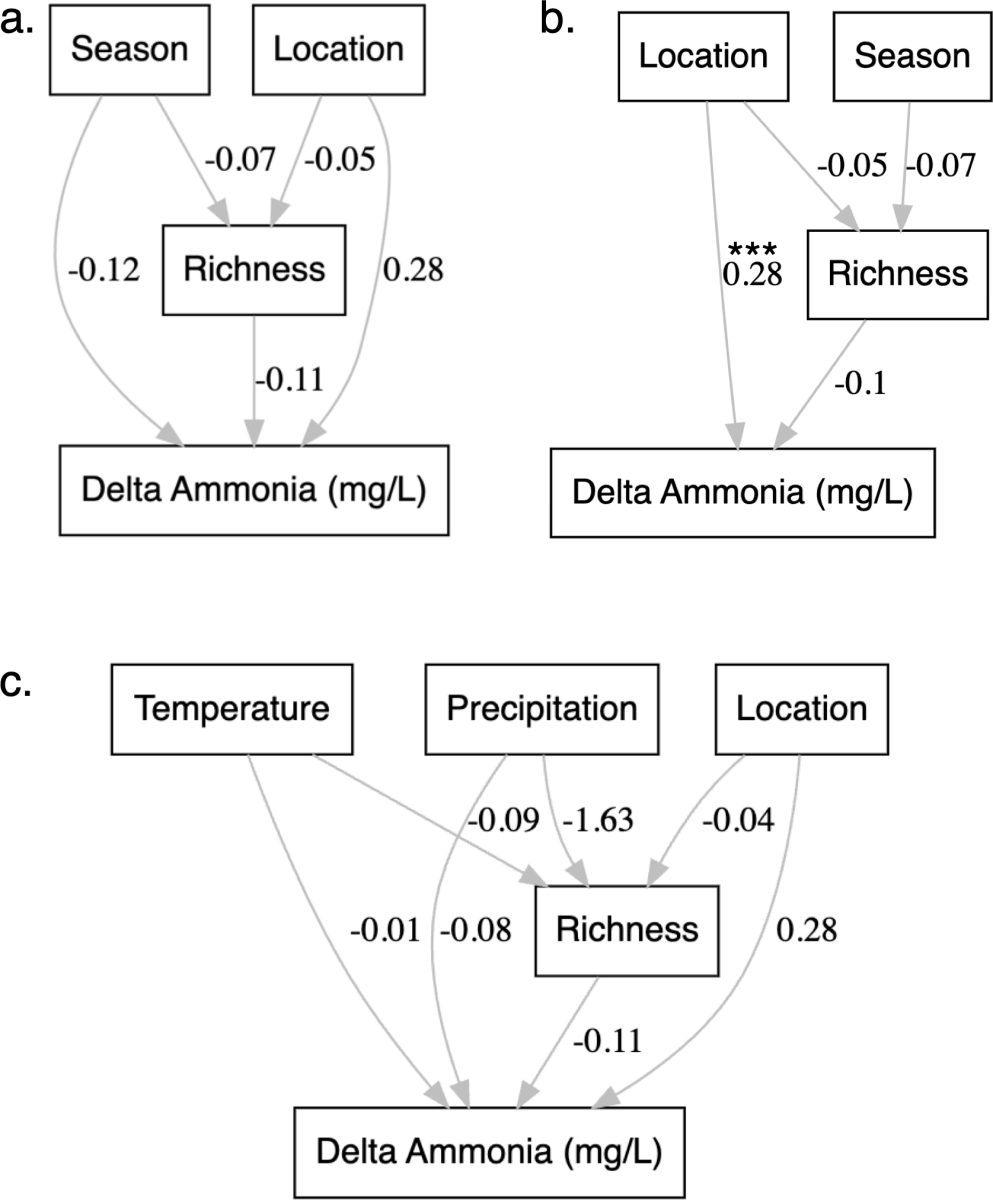
SEM illustrating hypothesized and simplified relationships between temporal and spatial factors on bacterial diversity and ammonia removal. We show (**a**) the *a priori* conceptual model, (**b**) the most parsimonious model retained based on information criteria, and (**c**) an exploratory model including temperature and precipitation. Arrows represent hypothesized directional relationships between measured variables (rectangles) and numbers correspond to standardized path coefficients. Asterisks (***) denote statistically significant paths (*P* < 0.001).

To better understand the influence of space and season on bacterial diversity and ammonia removal, while for BEF relationships, we used a SEM. Models consistently suggested a negative effect of richness on delta ammonia (Fig. 5), yet several of the most complex models were just-identified (saturated) due to the small sample size, precluding formal evaluation of model fit. For example, the full model including effects of season and location on richness and ammonia removal, as well as the BEF pathway (Fig. 5a), resulted in a saturated solution (χ² = 0, *P* = 0.000 CFI = 1.00, TLI = 1.00, RMSEA = 0), rendering global fit indices uninformative. We progressively simplified the model structure to obtain estimable models with positive degrees of freedom. Among these, the most parsimonious model based on AIC and BIC included direct effects of location and richness on ammonia removal, and season and location, for richness (Fig. 5b).

This model showed acceptable absolute fit (χ² (1) = 0.48, *P* = 0.49; RMSEA = 0.00; SRMR = 0.03). Incremental fit indices were high (CFI = 1.00; TLI = 1.20), reflecting improvement over the poorly fitting baseline model. In this model, location had a strongly significant positive effect on ammonia removal (β = 0.28, *P* < 0.001). Even though all parts of the best fit model, richness was not significantly associated with ammonia removal (β = −0.102, *P* = 0.473) and neither season (β = −0.067, *P* = 0.778) nor location (β = −0.047, *P* = 0.566) significantly predicted richness. We were not able to evaluate any of the models, including temperature or precipitation as exogenous factors, because they were saturated, but they consistently showed negative BEF relationships and a strong influence of precipitation over bacterial richness (Fig. 5c). Results should be interpreted cautiously given the small sample size (*n* = 33).

### Abiotic parameters associated with location and season

Temperature and precipitation seemed to be representatives of seasonal change, while total ammonia levels were closely related to sites along the series of treatment marshes. Temperature declined from autumn to winter, but did not change significantly with location (Fig. S2 top panels, *R*^2^ = 0.839, F_22,10_ = 8.589, *P* < 0.001). Precipitation followed a similar trend, increasing from autumn to winter and showing no response to location (Fig. S2 middle panels, *R*^2^ = 0.914, F_22,10_ = 14.29, *P* < 0.001). Because temperature and precipitation are weakly correlated (*R*^2^ = 0.78, F_1,31_ = 117.4, *P* < 0.001), we only test richness against one of the parameters, temperature, and find no significant relationship (Fig. S2 bottom panels, *R*^2^ = 0.05, F_1,31_ = 117.4, *P* = 0.687). Evenness followed the same trend and was unrelated to temperature (*R*^2^ = 0.05, F_1,31_ = 2.772, *P* = 0.106).

Total levels of ammonia declined throughout the system (Fig. S3) from oxidation pond 1 (25.08 mg/L) to the Bay Discharge (2.79 mg/L) with a slight peak at the EW influent (26.87 mg/L, fig ammonia). Overall, ammonia concentration ranged from 40 mg/L at oxidation pond 1 in the winter to 2.79 mg/L at the Bay discharge in the spring. The best-performing model included season as a fixed factor and location as a random factor (Table S5). Although the model was not significant after a Wald test (χ^2^ = 1.354, df = 2, *P* = 0.508), it was significantly better than the null model (χ^2^ = 25.055, df = 2, *P* < 0.01) and performed better than the linear model with season as fixed factor (Table S5). Unfortunately, pH, BOD, and DO measurements were compromised due to field conditions and technical difficulties; thus, they were not included in any further analysis.

### Bacterial composition and representative taxa

**Fig 6:**
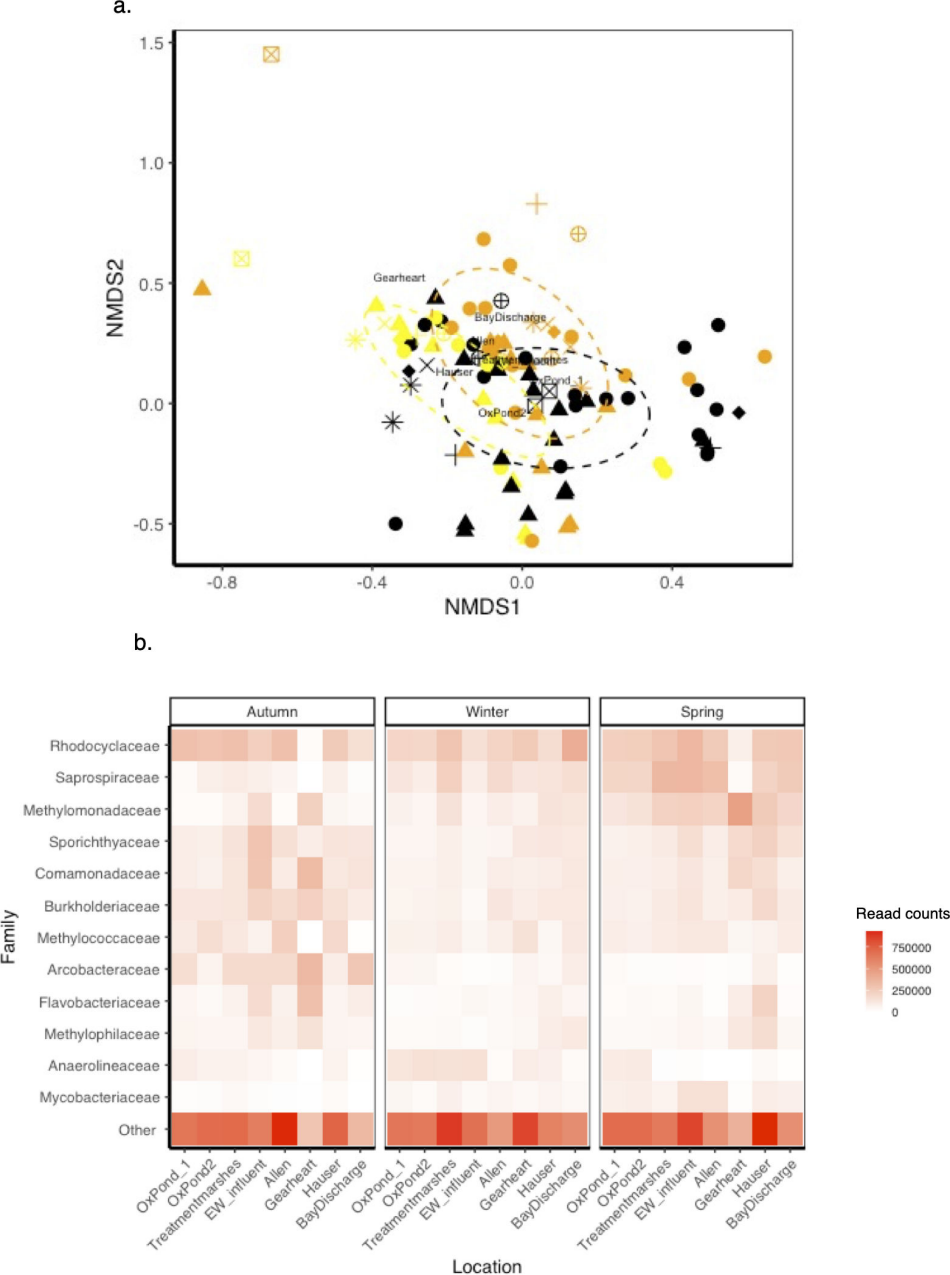
Community composition responses to location and season. (**a**) NMDS plot of ASV composition by season (color) and location (shape). The best solution for the NMDS had a stress of 0.1458 with a Procrustes RMSE of 0.010 and a maximum residual of 0.095. See Fig. 2 for legend details. Lines depict the covariance ellipses for the season. Centroids for location are indicated with the name of each pond. (**b**) Heatmap of average relative abundances at family-level taxonomic distribution. Shown are the 13 most abundant families in the data set with other taxa clustered in “others.” See Fig. S4 for an extended version of this figure. Darker tones indicate higher relative abundance.

Bacterial community composition responded to location and season (Fig. 6, perMANOVA: *R*^2^ = 0.306, F_23,89_ = 1.711, *P* = 0.001). The effect of Season was maintained after a pairwise test (F_2,89_ = 8.133, *P* = 0.02), but the effect of location was lost (F_7,105_ = 1.398, *P* = 0.098). Bacterial communities seemed more similar during spring than other seasons, even though we did not find significant differences (betadisper: F_2_= 2.877, *P* = 0.060). Location had no effect on community similarity (F_7_ = 1.099, *P* = 0.369).

Out of the original 346 ASVs, we found 11 taxa with positive influence on delta ammonia, 331 taxa with neutral or no association, and 3 taxa with negative association (Table S6). Taxa with positive associations with ammonia removal included those classified as *Legionella* sp*.* and Candidatus *Planktophila* sp*.* (Arcobacter) and belonging to Sulfurimonadaceae, Sporichthyaceae, Rhizobiales, Rhodocyclaceae, and Oxalobacteraceae. Taxa with a negative association with function included members of Omnitrophaceae, Methanosaetaceae, and Cloacimonadaceae. Most of these taxa’s abundances changed with location and season, except for Cloacimonadaceae, which only responded to season (Table S7). The qualitative analysis at the family level also revealed an interaction between location and season (Fig. 6b). Of the taxa identified above, only Rhodocyclaceae, Sporichthyaceae, and Arcobacteriaceae were numerically dominant (Fig. 6b). At the family level, three families were identified to have significant correlation with ammonia removal: Methylococcaceae, Burkholderiaceae, and Legionellaceae (Table S8). Some families increased in abundance with the season, including Mycobacteriaceae, Methylomonadaceae, and Saprospiraceae. A few groups decreased in abundance as seasons changed, including Rhodocyclaceae, Sporichthyaceae, Comamonadaceae, and Burkholderiaceae. Other groups varied from site to site and across seasons, making inferences challenging. For example, Arcobacter generally declined with season, but it was very variable across sites in autumn. Methylococcaceae was abundant in oxidation pond 2, Allen and Hauser marshes, but only in autumn samples, with a significant association with ammonia removal (Table S6). Notice that rare taxa are very abundant and vary across sites and seasons, highlighting the role of rare species.

Notably, Allen and Gearheart Ponds had the highest variability in family and genus composition, with a striking response to season. These ponds are lacking taxa that are otherwise abundant in the system, such as Rhodocyclaceae and Saprospiraceae in Gearheart Pond, and Methylomonadaceae and Flavobacteriaceae in Allen Pond, especially in autumn and spring (Fig. 6b). In contrast, these sites are enriched with taxa such as Methylococcaceae, Bulkholderiaceae, Comamonadaceae, Flavobacteriaceae, and Arcobacteraceae, particularly during autumn.

## DISCUSSION

A key goal in ecology today is to manage and sustain resilient ecosystem functions that persist in a human-influenced changing world (4, 105, 106). Steps forward should include a dynamic view of Biodiversity-Ecosystem function relationships that integrates abiotic and biotic processes shaping community diversity, composition, and functional outputs across space and time. We tested this framework in a constructed wetland used for wastewater secondary treatment and found a negative relationship between ammonia removal and bacterial diversity (Fig. 2). This pattern emerged from seasonal effects on bacterial diversity and location-specific influences on function (Fig. 3), highlighting the importance of considering the interaction between environmental, spatial, and biological factors. Our work thus adds to the growing body of research emphasizing the ecological context of BEF relationships (107, 108), which rarely account for spatial connectivity or report negative relationships.

Negative biodiversity-ecosystem relationships are rare in the literature (9, 18) and have primarily been documented for microbial degradation within aquatic (12, 13, 109, 110) and soil systems (111). These negative relationships are attributed to the negative selection effect, which proposes that species contributing most to ecosystem function are poor competitors, or subordinate species (11, 109), and are often members of a phylogenetically narrow group. Our findings suggest that bacteria with the highest potential for ammonia removal are unrelated subordinate species thriving in low-diversity locations after chlorination steps in the treatment train, supporting Hypothesis 1. The strong influence of chlorination on microbial communities (112) seems to be a disturbance point that overrides other aspects of connectivity and heterogeneity in this system. We propose that these disturbances create a refuge for bacterial taxa that support the highest ammonia removal rates in the system.

These disturbance points of ammonia removal refugia are clearly illustrated by the seasonal patterns in Allen and Gearheart ponds. These two sites have the lowest richness values (Fig. 3) and are lacking in taxa that are abundant otherwise in the system. They instead host taxa that are involved in critical steps in nitrogen cycling beyond just denitrification (Table 1), including Burkholderiaceae, Rhodocyclaceae, and Flavobacteriaceae. Chlorination steps may not only generate disturbance but also limit the dispersal of microorganisms from one pond to the next, minimizing community mixing. This limited dispersal can protect subordinate species from the arrival of antagonists, known as mass effects in metacommunity theory (27, 113). These patterns underscore the critical role of specific locations in facilitating ammonia removal from the system as functional refugia, which, to our knowledge, has not been reported in the context of wastewater treatment marshes. Notably, the effects of chlorination on Allen and Gearheart ponds are season-dependent, probably because the chlorination effect weakens during Humboldt’s wet winters, which bring higher flow rates into the AWTF, which dilute the impact of chlorination.

**TABLE 1:** Nitrogen cycle and bioremediation associated traits for the top seven most abundant taxa at the family level

Family	Relevant genus	Metabolic traits	Nitrogen cycle	Wastewater bioremediation	Reference
*Nanopelagicaceae*	Candidatus *Planktophila* sp.	Auxotrophic for amino acids, histidine pathway, and others.	Glutamine, glutamate synthesis, denitrification, nitrification	Potential for ammonia removal	(114–117)
*Burkholderiaceae*	*Cupriavidus* *Limnobacter* *Polynucleo-bacter*	Saprophytic Phyto-pathogen Opportunistic pathogens Toluene degradation	Denitrification Competitive acetate assimilation	Ammonia removal	(68, 118, 119)
*Flavobacteriaceae*	*Flavobacterium*	Diverse metabolic pathways and habitats	NA^*a*^	Chemotrophic breakdown of organic molecules	(120)
*Methylococcaceae*	*Methylonomas* *Methylococcus* *Methyloparacoccus*	Type-1 methano-trophs	Nitrogen fixation Nitrification	Methane oxidation	(121, 122)
*Methylomonadaceae*	*Methylospira*	Type-2 methanotrophs Anaerobic methane oxidation	Denitrification	Methane oxidation	(123–125)
*Rhodocyclaceae*	*Azonexus* *Propion-ividbrio*	Wide ranging	Denitrification Plant-associated nitrogen fixers	Fermentation Biodegradation of organic compounds	(126, 127)
*Sporichthyaceae*		Diverse carbohydrate sources	Nitrite uptake	Ammonia removal	(128)

^*a*^ NA, not applicable.

Seasonal change resulted in increased diversity during the wet winter months (Fig. 3) and had no significant effects on function (Fig. 2), thus refuting our Hypothesis 2. Instead, functional redundancy may explain this pattern, as taxa with similar functional roles replace one another as seasons shift, maintaining ammonia removal despite taxonomic turnover (62–64). For example, Burkholderiaceae, known competitive acetate assimilators during complete denitrification (118), decreased slightly from autumn to spring (Table S7) while several members of Methylococcaceae became abundant in spring and can play key roles in denitrification and methane fluxes, known as methanotrophic denitrification (129, 130). Additionally, macrophytes and algae can buffer seasonal change for bacteria by providing substrate and oxygen during winter months and carbon sources during summer months. We find a general negative BEF relationship with evidence of seasonal redundancy, suggesting redundancy can be context-dependent, perhaps contributing to an ongoing controversy regarding redundancy in microbial ecology. Traditionally, it was assumed that high diversity in microbial organisms implied functional redundancy (131), yet recent empirical evidence supports positive BEF relationships, suggesting complementarity (132, 133). Perhaps we should shift focus from one general BEF relationship and instead establish when to expect different BEF relationships based on ecological contexts and biological parameters.

Our SEM analysis revealed that bacterial richness had a stronger influence on ammonia removal than season or location (Fig. 5), underscoring the biological basis of this function as the primary mediator of environmental effects. The direct influence of location on ammonia removal suggests that site-specific conditions may impact per capita functioning or regulatory mechanisms, independent of bacterial population dynamics. Some of these site-specific effects include the impact of chlorination, the contribution of macrophytes to bacterial activity, and the declining ammonia levels across the treatment train. The role of per capita functioning in shaping ecosystem processes is understudied (Leander et al. 2016) and may be crucial to understand how environmental factors influence ecosystem functions like primary productivity (134) and nitrogen cycling (36). Our model also suggests season may have per-capita effects on function, while their direct impact on richness—linked to population shifts—appears weaker (Fig. 5). This combination of direct and indirect effects highlights a close and intricate interplay between environmental drivers, diversity, and functional output in wetland ecosystems. Our results align with previous work that integrates environmental context, site disturbances, and heterogeneity (but not connectivity) and species diversity into models that highlight the multifaceted drivers of ecosystem function (107, 135). In contrast, some studies have shown stronger environmental effects on microbial functionality than biodiversity (132, 136, 137), likely due to their focus on multifunctionality (138).

Although beyond the scope of this study, several additional factors may influence bacterial diversity and function in constructed wetlands that we did not address. First, dissimilatory nitrogen reduction to ammonia (DNRA) and nitrogen fixation are metabolic pathways that can maintain ammonia in marsh systems, contributing to the overall ammonia budget in wastewater treatment (139). Our system likely does not support important DNRA activity due to the declining organic load after oxidation pond 1 and low sulfide levels. Additionally, we did not detect large abundances of typical nitrogen-fixing taxa in our data set. Second, there are physicochemical pathways of nitrogen removal, such as ammonia volatilization (140, 141), that are not considered here, as our system’s low temperature and pH are unfavorable conditions for volatilization and observed rates of ammonia removal exceed those expected via this abiotic process (140).

Third, studies often emphasize the importance of plant-microbe interactions and biofilms in wetland water purification (142). Wetland rhizospheres create oxic-anoxic interfaces that stimulate nutrient cycling (143, 144). We acknowledge that plant and wildlife heterogeneity between ponds may influence nutrient cycling and bacterial diversity, but quantifying these variables was beyond the scope of this study due to technical constraints. Finally, sediment and detritus may contribute to increase ammonia in the system via the slow process of degradation and decay (52, 53), yet data from our system indicate sediment microbial communities are unaffected by space or season (71). We instead focused on the water fraction as a way to facilitate comparisons across varying vegetation, given that the volume of the aquatic habitat is larger and more impactful in our system. Although we do not have direct evidence of a connection between the microbial community and the ammonia levels, our findings are sufficiently robust to suggest a connection and coincide with findings from similar studies.

In fact, our findings coincide with previously reported ammonia levels, ammonia removal rates, bacterial diversity, and composition. We found ammonia levels that ranged from 2.79 to 40 mg/L and declined consistently as the water moved from the influx at oxidation pond 1 to the bay discharge point (Fig. S3). These results are comparable to those of constructed wetlands for domestic wastewater treatment reported before (38, 145). The community structure and dominant taxa we found were similar to previously reported work and included Rhodocyclaceae (146), Flavobacteriaceae (147–151), Saprospiraceae (72, 152, 153), Methylococcaceae (154), and many others (68) with critical functional roles (Table 1). Interestingly, one of the taxa with the strongest associations with ammonia removal, Candidatus *Planktophila* sp. (Actinobacteria, Table 1) is an unculturable auxotroph, hypothesized to participate actively in nitrogen cycling despite its small genome (115–117). *Planktophila* sp. has been recovered only when grown in a mixture because it requires amino acids and carbon metabolism intermediates (114). Procurement of such growth factors may be accomplished by *Legionella* sp. in our study, a taxon that was positively associated with function but with no evidence of nitrogen cycling functions. This highlights the role of species contributing to function and their critical interactions with other members of the community.

In contrast to the similarities outlined above, our findings deviate from some studies due to critical differences in hydraulic design (145, 148, 155–157), due to more extreme seasonal patterns (68, 93), focus on functions other than ammonia removal, and complex industrial or polluted influx (150, 158). It seems like the unique environmental, operational, and biological conditions of each wastewater treatment wetland result in case-specific management requirements and challenges (68).

Despite this individuality, three general recommendations stemming from our findings include (i) considering BEF theory when establishing relationships between biodiversity and purification functions, (ii) identifying functional refugia and focusing monitoring goals on those conditions, and (iii) recognizing functional redundancy and its buffering potential for climatic fluctuations. Wastewater treatment constructed wetlands represent a unique opportunity to apply and test our understanding of ecological processes and use microbial activity to reduce anthropogenic environmental impact. We propose that the concepts of functional refugia and seasonal functional redundancy may be useful in the context of a dynamic BEF field that recognizes the role of environmental context.

## Data Availability

Raw sequences are available via NCBI SRA with the accession number PRJNA1426766. Code scripts and metadata can be found on the CGLab GitHub page (https://github.com/catalicu/env_marsh_bef).

## SUPPLEMENTAL MATERIAL

Supplemental material spectrum.00229-25-s0001.docx Supplemental methods; Tables S1 to S5 and S7; Fig. S0 to S4

Table S6 spectrum.00229-25-s0002.xlsx Results from GLMs on relative abundance associations with function, for the most abundant 300 taxa.

Table S8 spectrum.00229-25-s0003.xlsx Results from GLMs on relative abundance associations with function, for the most abundant 13 Families.

ASM does not own the copyrights to Supplemental Material that may be linked to, or accessed through, an article. The authors have granted ASM a non-exclusive, world-wide license to publish the Supplemental Material files. Please contact the corresponding author directly for reuse.
