# Supplementary material for: Environmental effects on constructed wetland microbial diversity and function in the context of wastewater management
Source: Microbiol Spectr. 2026 Apr 21;14(6):e00229-25. doi: 10.1128/spectrum.00229-25 (PMC13227994; doi:10.1128/spectrum.00229-25)
Supplement: Supplemental material — Supplemental methods; Tables S1 to S5 and S7; Fig. S0 to S4 [file spectrum.00229-25-s0001.docx]

Title: Environmental effects on constructed wetland microbial diversity and function in the context of wastewater management

Authors: Sandrine Grandmont-Lemire^1^, Bob Gearheart^2^ and Catalina Cuellar-Gempeler^3^

**Supplementary material**

**Contents:**

1. **Supplementary Methods**
2. **Supplementary Tables**
3. **Supplementary Figures**
4. **Supplementary Methods**

The Arcata Wastewater Treatment Facility (AWTF)

The Facility was initially set up with a conventional Headworks System that removes large solids and organic material as the first treatment phase (Friends of the Arcata Marsh, Arcata Marsh Research Institute). Water is processed for less than a day before moving to the constructed wetlands. The Initial Oxidation ponds were set up in 1952 and, every day since then, an average of 5.9 million of gallons of water pass through an initial chlorination step and enters the secondary treatment in Oxidation pond 1 and 2 (Arcata Marsh Research Institute). With a depth of 3 m, water takes in average 45 days to move through each Oxidation pond since they cover approximately 40 acres of open water. Microscopic algae are the major photosynthetic organisms and UV exposure is estimated to be high since solids deposit quickly (Friends of the Arcata Marsh).

After the Oxidation ponds, water moves into the 6 parallel treatment wetlands (1-1.5m deep) where it takes water 1-5 days to circulate (Arcata Marsh Research Institute). These parallel treatment wetlands act as a progressive clarifier. Then, water goes through a second chlorination step before moving to the third phase.

The following enhancement ponds comprise the third phase and include Allen, Gearheart and Hauser ponds. These ponds were constructed in 1985 and cover 10 acres with an average depth of 1.5m (Friends of the Arcata Marsh). Complex habitats with aerobic and anaerobic zones result from a combination of open waters and vegetated areas through which water circulates. This portion of the Arcata Marsh is home to wildlife and a central feature of local outdoor activities. Water takes 20 days to circulate in the third phase before a last chlorination step and the Bay discharge point (Friends of the Arcata Marsh, Arcata Marsh Research Institute).

This system is purposedly separated from the bay by large barriers and there is no record of seawater intrusion. However, it is notable that constructed wetlands in coastal regions can often be exposed to seawater intrusion, and salinity has a negative effect on microbial metabolic activity as ammonia remover (Calheiros et al 2010, Belila et al 2016, Chen et al 2017, Fu et al 2019). Additionally, seasonal rains during the winter months (December to February) can dramatically increase the volume of flow diluting pollutants and microbial communities alike.

References

- Arcata Marsh Research Institute. Flow Path. Consulted on 10/28/2024 at https://arcatamarsh.wordpress.com
- Friends of the Arcata Marsh. Wastewater Treatment. Consulted on 10/28/2024 at https://www.arcatamarshfriends.org/the-marsh/wastewater-treatment/
- Calheiros CS, Teixeira A, Pires C, Franco AR, Duque AF, Crispim LF, Moura SC, Castro PM. 2010. Bacterial community dynamics in horizontal flow constructed wetlands with different plants for high salinity industrial wastewater polishing. Water Res 44(17):5032-8.
- Fu, Guiping, Jingyi Han, Tianyu Yu, Linkun Huangshen, and Lin Zhao. 2019. The structure of denitrifying microbial communities in constructed mangrove wetlands in response to fluctuating salinities. Journal of Environmental Management 238:1-9.
- Chen, Lijuan, Changsheng Li, Qi Feng, Yongping Wei, Hang Zheng, Yan Zhao, Yongjiu Feng, and Huiya Li. 2017. Shifts in soil microbial metabolic activities and community structures along a salinity gradient of irrigation water in a typical arid region of China. Sci. Total Environ. 598:64-70.
- Belila*et al.*2016. Bacterial community structure and variation in a full-scale seawater desalination plant for drinking water production. Water Res. 94:62-72.

1. **Supplementary Tables**

**Table S1. Seasonal temperature and precipitation for study period (2019-2020).** Averages, minimum and maximum daily values were calculated for each month from data obtained at the Eureka Woodley Island range gage station and the dataset managed by NOAA - National Center for Environmental Information ([www.ncdc.noaa.gov/cdo-web/datasets](http://www.ncdc.noaa.gov/cdo-web/datasets)). This standardized data was used to define Seasonal categories in our study.

**Table S2. Model selection to identify the role of richness (a) and evenness (b) as drivers of Ammonia removal.** Model comparisons and evaluation was conducted with AIC and BIC for richness (a) but only with AIC for evenness (b) since they both showed the same patterns.

| **(a) Richness Models** | **d.f.** | **AIC** | **BIC** |
| --- | --- | --- | --- |
| mod.null = lm(delta_ammonia ~ 1) | 2 | 126.865 | 129.523 |
| mod.rich.lm= lm(delta_ammonia ~ richness) | 3 | 123.617 | 127.614 |
| mod.rich.season.lm= lm(delta_ammonia ~ richness*Season) | 7 | 128.501 | 137.826 |
| mod.rich.season2.lm= lm(delta_ammonia ~ richness+Season) | 7 | 125.378 | 132.039 |
| mod.rich.location.lm= lm(delta_ammonia ~ richness * Location) | 15 | 115.191 | 135.174 |
| mod.rich.location2.lm= lm(delta_ammonia ~ richness+Location) | 9 | 116.944 | 128.934 |
| **mod.rich.fix= lm(delta_ammonia ~ richness*Season+Location)** | **17** | **112.665** | **135.313** |
| mod.rich.fix2= lm(delta_ammonia ~ richness*Location+Season) | 13 | 119.370 | 136.688 |

| **(b) Evenness Models** | **d.f.** | **AIC** |
| --- | --- | --- |
| mod.null = lm(delta_ammonia ~ 1) | 2 | 126.865 |
| mod.rich.lm= lm(delta_ammonia ~ eveness) | 3 | 126.182 |
| mod.rich.season.lm= lm(delta_ammonia ~ eveness *Season) | 7 | 131.996 |
| **mod.rich.season.lm= lm(delta_ammonia ~ eveness *Location)** | **15** | **91.059** |
| mod.rich.season.lm= lm(delta_ammonia ~ eveness *Season+Location) | 13 | 116.315 |
| mod.rich.season.lm= lm(delta_ammonia ~ eveness *Location+Season) | 17 | 91.438 |

**Table S3. Model selection to identify the direct role of location and season on Ammonia removal.** Selected model based on AIC is shown in bold.

|  | **d.f.** | **AIC** |
| --- | --- | --- |
| mod.null = lm(delta_ammonia ~ 1) | 2 | 168.6392 |
| mod.am.rand = lm(delta_ammonia ~ Location | 9 | 130.2704 |
| mod.am.season.lm= lm(delta_ammonia ~ Season) | 4 | 172.5924 |
| **mod.am.season.lmei= lm(delta_ammonia ~ Season * Location** | **24** | **89.5773** |

**Table S4. Model selection to identify the direct role of location and season on ASV richness (a) and evenness (b).** Selected model based on AIC is shown in bold.

| **(a) Richness Models** | **d.f.** | **AIC** |
| --- | --- | --- |
| mod.null = lm(richness ~ 1) | 2 | 536.8255 |
| mod.rich.rand= lmer(richness ~ 1 + (1\|Location) | 3 | 530.1811 |
| mod.rich.season.lm= lm(richness ~ Season) | 4 | 532.8830 |
| **mod.rich.season.lmei= lm(richness ~ Season + (1\| Location)** | **5** | **506.0260** |

| **(b) Evenness Models** | **d.f.** | **AIC** |
| --- | --- | --- |
| mod.null = lm(evenness ~ 1) | 2 | -117.68857 |
| mod.eve.rand= lmer(evenness ~ 1 + (1\|Location) | 3 | -107.97008 |
| **mod.eve.season.lm= lm(evenness ~ Season)** | **4** | **-125.20348** |
| mod.eve.season.lmei= lm(evenness ~ Season + (1\| Location) | 5 | -102.74825 |

**Table S5. Model selection to identify the direct role of location and season on total ammonia concentrations.** Selected model based on AIC is shown in bold.

|  | **d.f.** | **AIC** |
| --- | --- | --- |
| mod.env.null = lm(total_ammonia ~ 1) | 2 | 242.5508 |
| mod.env.rand = lmer(total _ammonia ~ 1 + (1\|Location) | 3 | 231.8745 |
| mod.env.season.lm= lm(total _ammonia ~ Season) | 4 | 246.0497 |
| **mod.am.season.lmei= lm(total_ammonia ~ Season + (1\| Location)** | **5** | **226.5119** |

**Table S6. Results from GLMs on relative abundance associations with function, for the most abundant 300 taxa.** Taxa were clustered at the ASV taxonomic level. See excel supplementary table with tabs for taxonomic level (TableS6.xlsx).

**Table S7. ASV relative abundance responses to space and time for selected taxa.** Taxa were clustered at the ASV taxonomic level.

| **Top performing model** | **Chisq** | **df** | **pval** | **adj.p** | **Family** | **Genus** |
| --- | --- | --- | --- | --- | --- | --- |
| lmer(ASVrelabun ~ Season + (1\|Location)) | 7.677 | 2 | 0.021 | 0.059 | Sporichthyaceae | NA |
| lmer(ASVrelabun ~ Season + (1\|Location)) | 9.644 | 2 | 0.008 | 0.044 | Sporichthyaceae | Candidatus Planktophila |
| lmer(ASVrelabun ~ Season + (1\|Location)) | 1.598 | 2 | 0.449 | 0.70 | Oxalobacteraceae | NA |
| lmer(ASVrelabun ~ Season + (1\|Location)) | 7.086 | 1 | 0.007 | 0.044 | Rhodocyclaceae | C39 |
| lmer(ASVrelabun ~ Season + (1\|Location)) | 0.713 | 2 | 0.699 | 0.769 | Sporichthyaceae | Candidatus Planktophila |
| lmer(ASVrelabun ~ Season + (1\|Location)) | 4.986 | 2 | 0.082 | 0.181 | Legionellaceae | Legionella |
| lmer(ASVrelabun ~ Season + (1\|Location)) | 1.240 | 2 | 0.537 | 0.712 | Sporichthyaceae | Hgcl clade |
| lmer(ASVrelabun ~ Season + (1\|Location)) | 7.999 | 2 | 0.018 | 0.05 | Sulfurimonadaceae | Sulfurimonas |
| lmer(ASVrelabun ~ Season + (1\|mock)) | 1.881 | 2 | 0.390 | 0.706 | PBS-18 | NA |
| lmer(ASVrelabun ~ Season + (1\|Location)) | 0.520 | 2 | 0.770 | 0.770 | Sulfurimonadaceae | Sulfuricurvum |

**Table S8. Results from GLMs on relative abundance associations with function, for the most abundant 13 Families.** The model included relative abundance as fixed factor and location as a random factor. Taxa were clustered at the Family taxonomic level. See excel supplementary table with tabs for taxonomic level (TableS8.xlsx).

1. **Supplementary Figures**

**
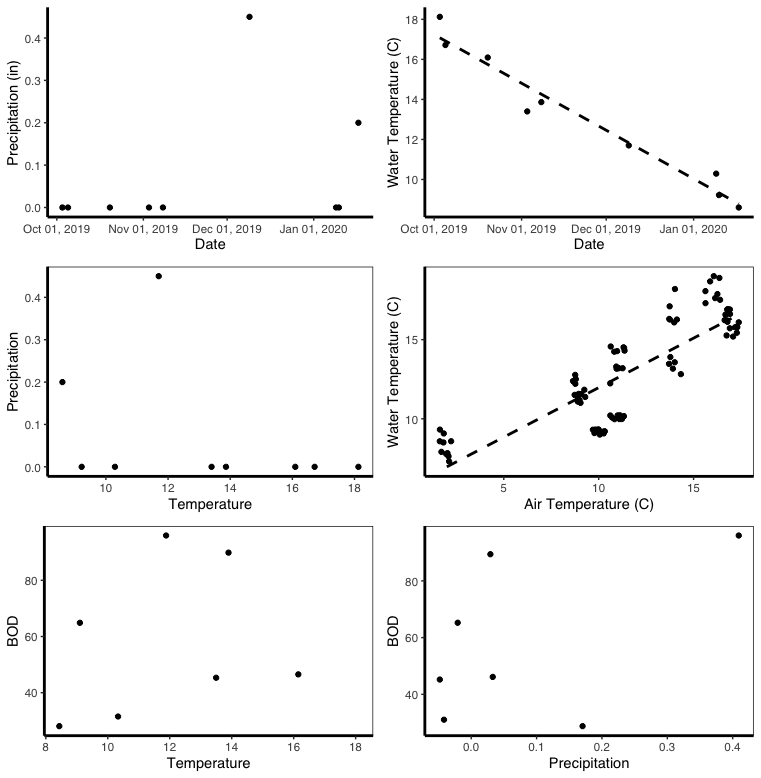
**

**Figure S0.** Environmental correlations. Data was averaged per date for these analyses to account for pseudo replication. An exception was made for temperature comparisons (center right panel), because coupled measurements were made at all times. Significant correlations are shown in dashed lines between date and water temperature (top right, F_1,7_=163.4, p<0.001, adjusted R^2^=0.953), and between water temperature and air temperature (center right, F_1,92_=215.7, p<0.001, adjusted R^2^=0.697).


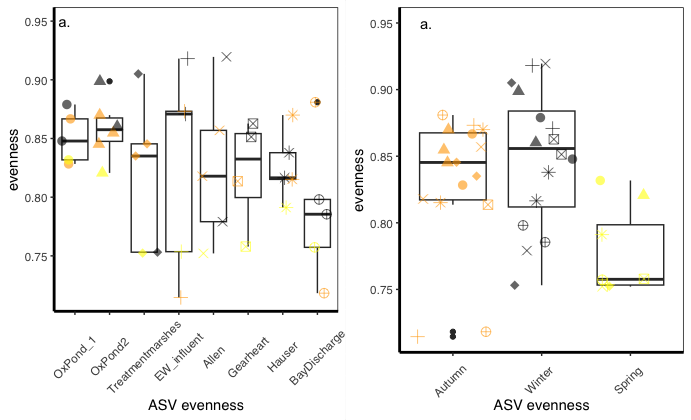


**Figure S1.** ASV eveness responses to a) location and b) season. Datapoints represent individual samples at each sampling event. Colors represent Seasons in (a) while shapes represent locations in (b). Oxidation pond data was averaged to account for their large sizes and multiple sampling.


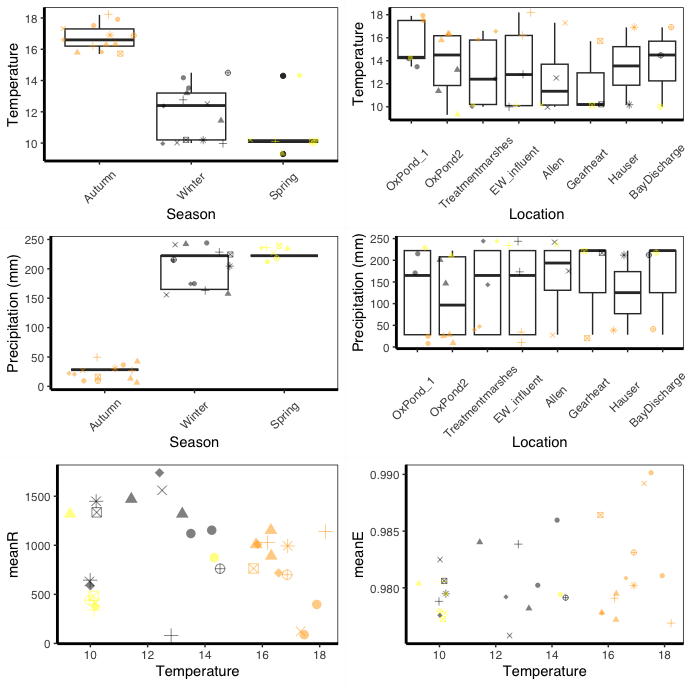


**Fig S2.** Environmental parameters by season (left) and location (right). Temperature (top panels) and precipitation (middle panels) data are represented with boxplots summarizing distributions and points showing raw datapoints. Relationship between temperature and mean richness (bottom left) and mean evenness (bottom right). Colors (season) and shapes (location) are as described in Fig 2 in the main text.


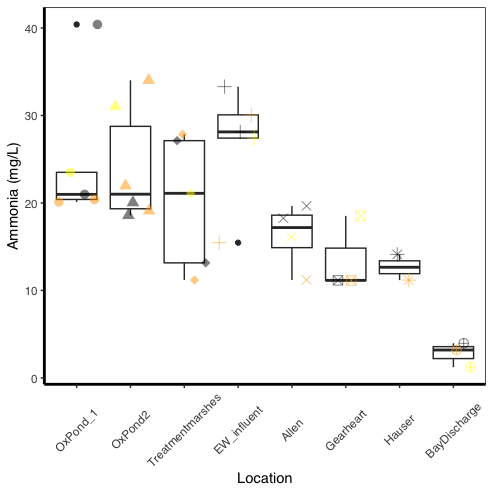


**Fig S3.** Ammonia concentration (mg/L) declines from site to site following the treatment train from the facility influent to the Humboldt Bay discharge point. Colors (season) and shapes (location) are as described in Fig 2 in the main text.


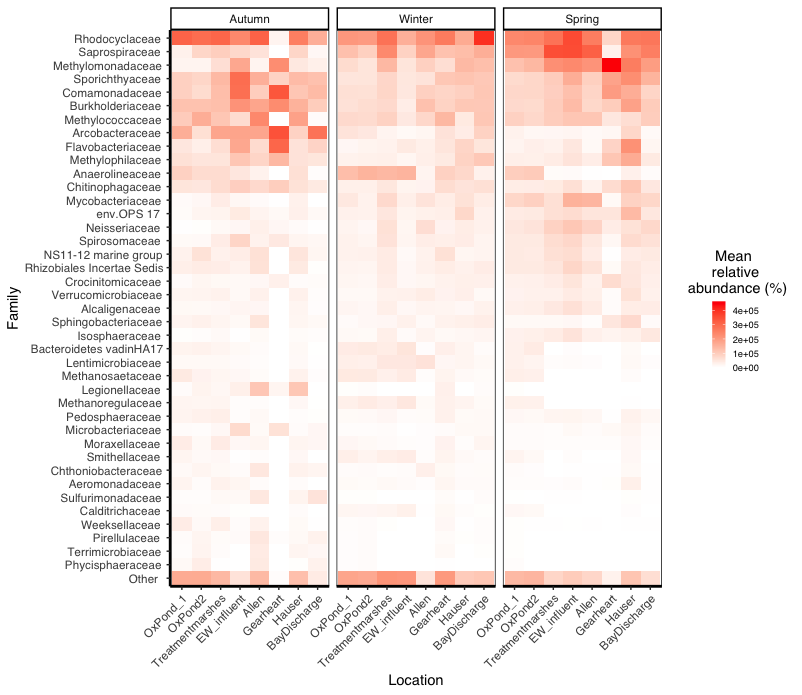


Figure S4. Heatmap displaying the relative abundance of microbial taxa over seasons and locations. Data is clustered at the Family taxonomic level. Color intensity in each panel shows the percentage in a sample, referring to the color key on the side.
